# Metformin Affects the Transcriptomic Profile of Chicken Ovarian Cancer Cells

**DOI:** 10.3390/genes13010030

**Published:** 2021-12-23

**Authors:** Lalitha Gopalan, Aswathy Sebastian, Craig A. Praul, Istvan Albert, Ramesh Ramachandran

**Affiliations:** 1 Department of Animal Science, The Pennsylvania State University, University Park, PA 16802, USA; lxg254@psu.edu; 2 The Huck Institutes of the Life Sciences, The Pennsylvania State University, University Park, PA 16802, USA; azs13@psu.edu (A.S.); cap142@psu.edu (C.A.P.); iua1@psu.edu (I.A.); 3 Department of Biochemistry and Molecular Biology, The Pennsylvania State University, University Park, PA 16802, USA; 4 Center for Reproductive Biology and Health, Department of Animal Science, The Pennsylvania State University, University Park, PA 16802, USA

**Keywords:** transcriptomics, ovarian cancer, metformin, animal models

## Abstract

Ovarian cancer is the most lethal gynecological malignancy in women. Metformin intake is associated with a reduced incidence of ovarian cancer and increased overall survival rate. We determined the effect of metformin on sphere formation, extracellular matrix invasion, and transcriptome profile of ovarian cancer cells (COVCAR) isolated from ascites of chickens that naturally developed ovarian cancer. We found that metformin treatment significantly decreased sphere formation and invasiveness of COVCAR cells. RNA-Seq data analysis revealed 0, 4, 365 differentially expressed genes in cells treated with 0.5, 1, 2 mM metformin, respectively compared to controls. Transcriptomic and ingenuity pathway analysis (IPA) revealed significant downregulation of *MMP7*, *AICDA*, *GDPD2*, *APOC3*, *APOA1* and predicted inhibition of upstream regulators *NFKB*, *STAT3*, *TP53* that are involved in epithelial–mesenchymal transition, DNA repair, and lipid metabolism. The analysis revealed significant upregulation of *RASD2*, *IHH*, *CRABP-1* and predicted activation of upstream regulators VEGF and E2F1 that are associated with angiogenesis and cell cycle. Causal network analysis revealed novel pathways suggesting predicted inhibition of ovarian cancer through master regulator *ASCL1* and dataset genes *DCX*, *SEMA6B*, *HEY2*, and *KCNIP2*. In summary, advanced pathway analysis in IPA revealed novel target genes, upstream regulators, and pathways affected by metformin treatment of COVCAR cells.

## 1. Introduction

Ovarian cancer is the most lethal gynecological malignancy in women. The American Cancer Society estimates that approximately 21,140 new cases of ovarian cancer will be diagnosed, and about 13,770 women are likely to die due to this disease in the United States in the year 2021. Even though significant progress has been made in ovarian cancer treatment, the 5-year survival rate of ovarian cancer remains less than 50% [1]. The higher rate of mortality is attributed to the fact that women with ovarian cancer do not exhibit symptoms at an early stage but are often (>85%) diagnosed at advanced stages of disease progression resulting in a poor (27%) 5-year survival rate. Metformin is a biguanide drug used in the treatment of T2DM since 1957 in Europe and since 1995 in USA [2].

In addition to its anti-diabetic effect, metformin has gained attention for its pleiotropic beneficial effects, including but not limited to reduced cancer risk [3], tumorigenesis [4] and improved overall survival in ovarian cancer patients [5]. A case-control analysis from a United Kingdom-based general practice database revealed that long-term use of metformin was associated with a reduced risk of ovarian cancer [6]. In a phase II clinical trial of metformin as neoadjuvant to chemotherapy and/debulking surgery patients had a median progression-free-survival (PFS) of 18.3 months and overall survival (OS) of 58.0 months for those with stage IIC/III EOC, while patients in stage IV EOC had a median PFS of 14.8 months and OS of 22 months. In the same study, in addition to metformin having a beneficial effect on PFS and OS, metformin-treated tumors had 2.4-fold decrease in ALDH+CD133+ cancer stem cells (CSCs) and increased sensitivity to cisplatin ex vivo [7]. A single-institution retrospective study of 341 ovarian cancer patients evaluated the effect of metformin on PFS and OS. The PFS at 5-years for diabetic patients who used metformin was 51% compared to 23% for patients without diabetes and 8% fro diabetic patients who did not use metformin [8]. Another meta-analysis study of metformin intake in diabetic patients focused on incidence and prognosis for ovarian cancer patients, had a pooled odds ratio of 0.76 (95% CI, 0.62–0.93, *p* = 0.008) for incidence and 0.55 (95% CI 0.36–0.84, *p* = 0.006) for prognosis [9]. A comparison study in Taiwanese women with ovarian cancer showed an overall adjusted hazard ratio (95% confidence interval) of 0.658 for metformin ever-users versus metformin never-users [10].

Furthermore, metformin treatment has shown to affect ovarian cancer cells derived from rodent models of ovarian cancer and in human ovarian cancer cell lines [11,12,13,14]. An understanding of the anti-cancer effects of metformin in clinical trials involving ovarian cancer is challenging since the administration of metformin is typically done in conjunction with chemotherapeutic drugs, debulking surgery, or both. Therefore, in vitro studies using primary ovarian cancer cells to elucidate the anti-cancer effects of metformin are essential. In addition to using patient-derived primary ovarian cancer cells, the use of primary ovarian cancer cells obtained from animal models that develop ovarian cancer naturally can facilitate our understanding of the anti-cancer effect of metformin.

Animal models that develop ovarian cancer spontaneously can be instrumental in understanding the etiology, progression, and therapy that are difficult to study in humans [15,16]. The laying hen model is the only animal model that develops ovarian cancer spontaneously and naturally as it occurs in women [17,18,19,20]. Although the etiology of ovarian cancer in chickens is unknown, incessant ovulation [21] and mutations in tumor suppressor protein p53 (TP53), RAS, and human epidermal growth factor-2 (HER) receptor [22] contribute to the high rate of ovarian cancer incidence, similar to etiology of OC in women. A high proportion 24–40% of hens aged between 2 and 4 years develop ovarian epithelial adenocarcinoma [23]. Our laboratory isolated ovarian cancer cells from ascites of chickens (COVCAR) and found that the COVCAR cells expressed several genes and proteins typically associated with ovarian adenocarcinoma and ovarian cancer cell lines derived from human subjects [23]. We also found that the aldehyde dehydrogenase 1A-positive (ALDH+) stem cells isolated from the ascites of chickens that developed ovarian cancer readily formed sphere in three-dimensional cell culture and were highly invasive in extracellular matrix proteins compared to ALDH- cells [24].

The hypothesis of our study is that metformin causes inhibition of sphere formation, decreased Matrigel invasion and affects the transcriptomic profile of ovarian cancer cells. The objectives of the present study are to determine the effects of metformin on sphere formation, Matrigel invasion, and the transcriptome of COVCAR cells. The research presented here is the first report to examine the transcriptomic profile of spheres formed by metformin-treated primary ovarian cancer cells. Our results suggest that metformin treatment affects sphere formation and invasiveness that are accompanied by significant changes in transcriptomic profile of genes related to epithelial–mesenchymal transition (EMT), cell proliferation, lipid synthesis, apoptosis, cell cycle progression, and angiogenesis.

## 2. Materials and Methods

### 2.1. Animal, Isolation of COVCAR Cells

All animal procedures were conducted according to the Institutional Animal Care and Use Committee (IACUC) approved protocol. Leghorn chickens (Hy-Line W-36 strain), which were past their peak production age (2–4 years old), were reared at the Poultry Education and Research Center at The Pennsylvania State University (University Park, PA, USA). Chickens were raised in wire cages under a photo-period of 16 h light:8 h dark and provided ad libitum feed and water.

### 2.2. Primary Ovarian Cancer Cell Culture

Chickens were routinely screened for clinical symptoms such as distended abdomen, pale comb, and inanition. Chickens suspected of having ovarian cancer were euthanized by cervical dislocation and ascites was collected from four chickens (*n* = 4) maintaining aseptic conditions. Ascites fluid was centrifuged at 400× *g* for 10 min and the resultant cell pellet were resuspended in MCDB 105:M199(1:1) culture medium (Sigma-Aldrich, St. Louis, MO, USA) supplemented with 20% fetal bovine serum (Atlanta Biologicals, Atlanta, GA), MEM amino acid (Cellgro-25-030-CI), glutamine (Glutamax, Gibco), sodium pyruvate (Sigma Aldrich, St. Louis, MO, USA), l-alanine l-glutamine dipeptide (Cellgro), Anti-Anti (100×, Gibco), glucose (GIBCO-15023-021). COVCAR cells were cultured on gelatinized 75 cm${}^{2}$ flasks at 37 ${}^{\circ }$C under 5% CO_2_ atmosphere. When the COVCAR cells reached 100% confluence, the cells were dissociated from the flask using 0.05% trypsin (Invitrogen). COVCAR cells were frozen in several aliquots and stored in the vapor phase of liquid nitrogen and used for further experimentation. Frozen COVCAR cells in their early passage (1–2 passages) were thawed and used for the following experiments.

### 2.3. Effect of Metformin on COVCAR Cell Sphere Formation

Approximately 200,000 COVCAR cells from each of 4 primary ovarian cancer lines designated as C5, C7, C9, and C11 were treated with 0, 0.5, 1, or 2 mM metformin (Sigma-Aldrich) dissolved in serum-free medium [SCM; X-VIVO 20 culture medium (Lonza, Walkersville, MD, USA) supplemented with 5 μg/mL bovine insulin (Sigma-Aldrich), 20 ng/mL recombinant human epidermal growth factor (Peprotech, Rocky Hill, NJ, USA) and antibiotic-antimycotic solution (Gibco)] in 24-well ultra-low attachment plate (Corning, NY, USA) for 24 h at 37 ${}^{\circ }$C under 5% CO_2_ atmosphere. Spheres were visualized using a Zeiss microscope (Carl Zeiss, New York, NY, USA) and photographed using Axiocam digital camera (Carl Zeiss) to determine sphere count. The total number of spheres greater than 50 μm at 10× magnification from three non-overlapping fields were counted and averaged. A total of 6-wells/treatment served as internal replicate. Experiments with each cell line were performed in triplicate.

### 2.4. Effect of Metformin on Matrigel Invasion of COVCAR Cells

COVCAR cells (C5, C7, C9, and C11) were treated with metformin and subjected to Matrigel invasion assay. The upper chamber of transmembrane cell culture inserts (Corning, NY, USA) having 8 μm pores was coated with 200 μL Matrigel extracellular matrix (2 mg/mL; CORNING, NY] in serum-free SCM and allowed to solidify at 37 ${}^{\circ }$C under 5% CO_2_ for 1 h. Approximately 150,000 cells were placed into each 12 × 75 mm polypropylene tube (VWR, Radnor, PA, USA) and treated with 0 (control), 0.5, 1, or 2 mM metformin and allowed to incubate in a shaking water bath at 37 ${}^{\circ }$C for 30 min. Metformin-treated cells were then carefully layered over the Matrigel-coated inserts. Chemoattractant media (SCM with 20% FBS) was added to the lower chamber of the transmembrane cell culture inserts before placing the inserts. Cells were allowed to invade through the Matrigel layer at 37 ${}^{\circ }$C under 5% CO_2_ for 24 h. Following incubation, cells remaining in the Matrigel layer on the upper surface of the inserts were removed using cotton swabs. Cells that had invaded the Matrigel layer and reached the lower surface of the insert were fixed in methanol and stained with Giemsa stain. The invaded cells were visualized at 10–20× magnification using a Zeiss microscope (Zeiss) and photographed using Axiocam digital camera (Zeiss). The number of cells was counted and averaged from 4 non-overlapping fields per insert. The entire experiment was repeated six times using all four cell lines.

Data from sphere formation assay and Matrigel invasion assay were subjected to analysis of variance (ANOVA) using the general linear model (GLM) using R software and GraphPad Prism version 8.0.0. Differences among individual means were determined by using Tukey’s HSD test. A probability level of *p*-value > 0.05 was considered statistically significant.

### 2.5. Effect of Metformin on COVCAR Cell Transcriptome

COVCAR cells designated as C5, C7, C9, and C11 (*n* = 4) were utilized for transcriptomic analyzes. Approximately 300,000 cells were treated with 0 (control), 0.5, 1, or 2 mM metformin dissolved in SCM in 24-well ultra-low attachment plates (Corning) at 37 ${}^{\circ }$C under 5% CO_2_ for 24 h. Each treatment was conducted in triplicate. Cells and spheres following the treatment were harvested by centrifugation followed by resuspension in TRIzol (Invitrogen) and stored at −80 ${}^{\circ }$C until RNA extraction. Total RNA was extracted using the RNeasy Micro kit (Qiagen, Valencia, CA, USA) using the manufacturer’s protocol. The quantity and quality of RNA were evaluated using a spectrophotometer (Nanodrop, Wilmington, DE, USA). The RNA quality was further evaluated using the Agilent TapeStation 4150 (Agilent Technologies, Santa Clara, CA, USA). Samples that had an RNA integrity number (RIN) value of >7 were used for sequencing. RNA sequencing was performed on the Illumina platform using 200 ng of total RNA as input. A unique dual indexed library was made from each sample using the TruSeq Stranded mRNA Library Prep Kit according to the manufacturer’s protocol (Illumina, San Diego, CA, USA). The concentration of each library was measured by qPCR using the KAPA Library Quantification Kit for Illumina platforms (KAPA Biosystems, Wilmington, MA, USA). An equimolar pool was made from all libraries and the pool was sequenced on two NextSeq 550 High Output 75-nucleotide single read sequencing runs.

On average, 50 million 75-base pair single-end reads were sequenced for each replicate. FastQC reports showed that the raw reads have a mean base quality score of Q35. Raw data were mapped to the chicken reference genome (Gallus [underscore] gallus. GRCg6a build) using hisat2 (v2.1.0). Coverage files were generated using bedtools and the mapping was visualized in Integrative Genomics Viewer (IGV). The average mapping rate was 94% of the Gallus gallus genome. Reads mapping to the genes were counted using featureCounts from the Subread package specifying ‘-t exon -s 2 -O -g geneid’ parameters. Statistical analysis to identify differentially expressed genes (DEGs) based on negative binomial distribution was conducted using R (v. 4.1.0.) package DESeq2.

### 2.6. Data Visualization

GProfiler was used to evaluate the biological processes enhanced in the set of DEGs. Galaxy version 21.05.1.dev0 was used to create volcano plots. We used IPA to identify canonical pathways, upstream regulators, gene networks, diseases, and functions affected by metformin treatment. The statistical significance of pathways was determined using *p*-value, and the z-score, which provides predictions about upstream or downstream processes. A positive z-score of 2 or more indicates a significant activation and a negative z-score of 2 indicates a significant inhibition of a pathway, regulator, or disease process. Casual network analysis (CNA) was performed in Ingenuity pathway analysis (IPA) by considering the following: (i) upstream regulator analysis (URA), which connects transcription factors, cytokines, groups, microRNAs to dataset molecules; (ii) mechanistic networks (MN) involving the greatest number of dataset molecules and MN that connects to transcription factors; (iii) causal relationships that connect upstream regulators to dataset molecules through intermediate regulators; and (iv) downstream effect analysis (DEA) that utilizes the URA and CNA to make an inference of downstream biological processes and diseases. We utilized IPA’s canonical pathways to show the key molecules and biological processes involved in a certain the pathway. In addition, we analyzed canonical pathways by using molecular activity predictor (MAP) and dataset molecules/analysis overlay function.

## 3. Results

### 3.1. Effect of Metformin on Sphere Formation and Matrigel Invasion

**Figure 1:**
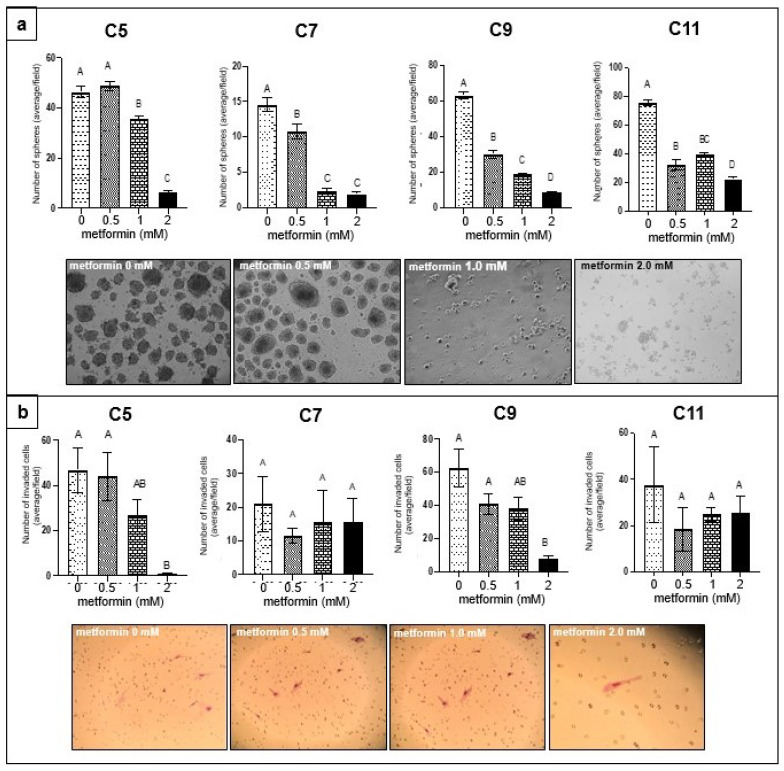
Effect of metformin on ovarian cancer cells (**a**) Sphere formation assay: COVCAR cell lines (C5, C7, C9, C11) were treated with 0, 0.5, 1, or 2 mM metformin for 24 h in ultra-low attachment culture wells. The average number of spheres from 6 wells was calculated for each metformin concentration tested. The experiments were performed in triplicates. [A–B, *p* < 0.05; A–C, *p* < 0.01; A–D, *p* < 0.001; B–C, *p* < 0.05; C–D, *p* < 0.05]. (**b**) Matrigel invasion assay: COVCAR cells were treated with 0, 0.5, 1, 2 mM metformin for 30 min and were then allowed to invade through Matrigel for 24 h. The total number of invaded cell at the bottom of well were counted. Experiments were performed in triplicate. (A–B) *p* < 0.05. Inset: photomicrographs of spheres and cells invaded through Matrigel.

We evaluated the effect of metformin treatment on sphere formation in four (C5, C7, C9, and C11) primary COVCAR cell lines. Metformin treatment at at 1-mM and 2-mM led to significantly less spheres in all four cell lines as compared to the control group (Figure 1). Metformin treatment of C5 and C9 COVCAR cell lines resulted in significantly fewer number of cells that invaded Matrigel (Figure 1).

### 3.2. Transcriptomic Analysis of Metformin-Treated COVCAR Cells

**Figure 2:**
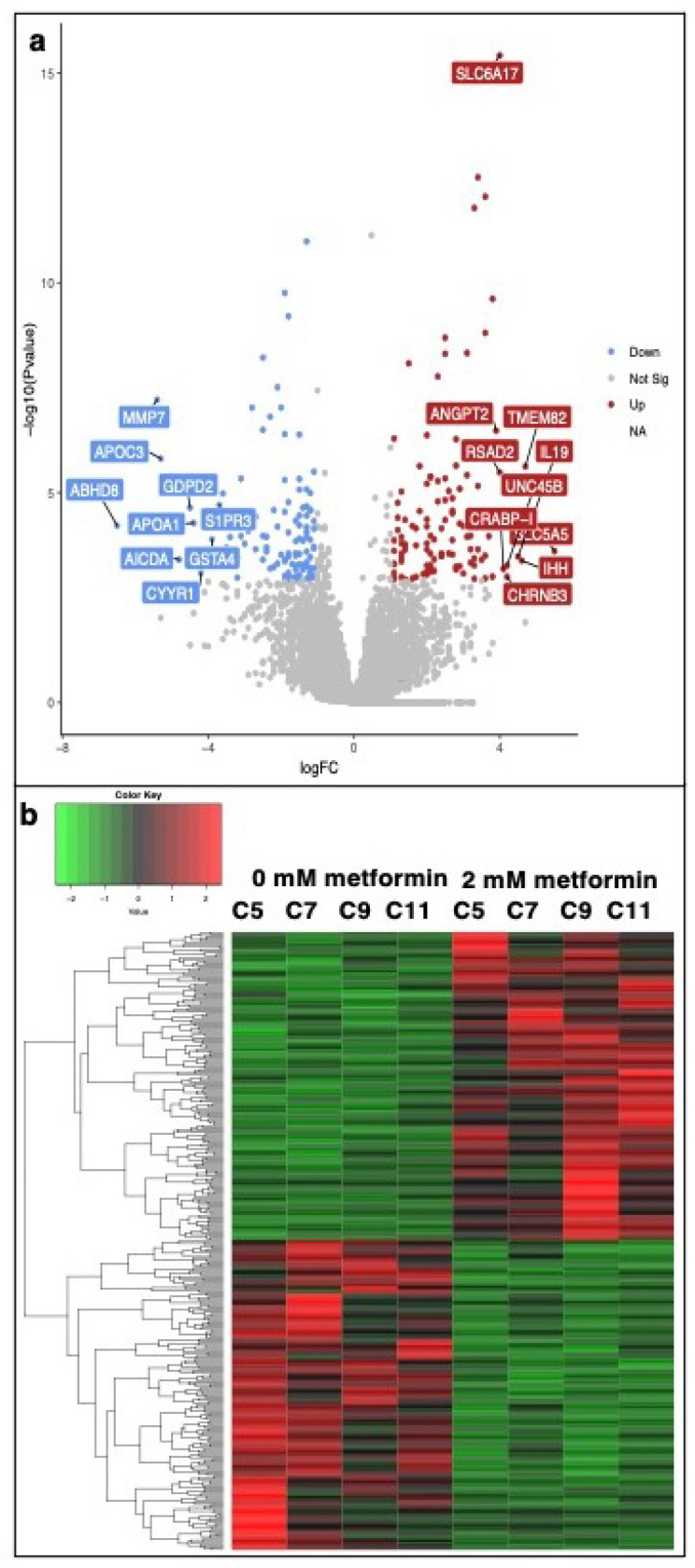
RNASeq analysis of mRNA extracted from metformin-treated COVCAR cells. COVCAR cell lines (C5, C7, C9, C11) were treated with 0, 0.5, 1, or 2 mM metformin for 24 h in ultra-low attachment culture wells. Total RNA was extracted and subjected to RNA sequencing Illumina sequencing. Statistical analysis was performed using DESeq2 in R v.4.1.0. to identify the differentially expressed genes (DEGs). (**a**) Volcano plot to indicate the differentially expressed genes (DEGs). Colored for |log2FC| > 1 and FDR < 0.05. (**b**) Heatmap of hierarchically clustered 365 DEGs between 0- and 2-mM metformin-treated COVCAR cells.

**Figure 3:**
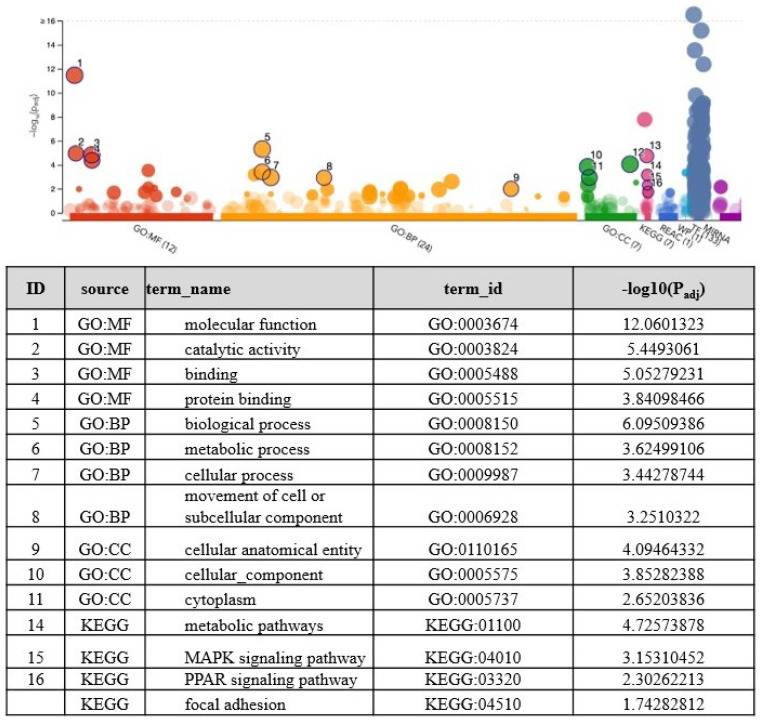
Manhattan plot created with g: GOSt in g: Profiler that illustrates enrichment of gene ontology (GO) terms associated with DEGs between 0- and 2-mM metformin-treated COVCAR cells: the x-axis represents functional terms that are grouped and color-coded. From GO: molecular function (MF), biological process (BP), cellular components (CC), and KEGG pathways. The color intensity denotes the significance of GO terms. The y-axis shows the adjusted enrichment *p* values in −log10 (Padj). The table under the Manhattan plot identifies the top GO terms associated with MF, BP, CC, and KEEG pathways and are numbered by −log10 (Padj) values.

Pairwise comparison analysis using DESeq2 revealed a dose-dependent increase in the number of DEGs in response to metformin treatment. We found that 0, 4, 365 genes (*p* < 0.01, FDR < 0.05) were differentially expressed in cells treated with 0.5, 1, or 2 mM metformin compared to the control group, respectively (Supplementary Table S1). The top 10 upregulated and downregulated DEGs along with their biological functions are provided in Table 1 and Table 2. A graphical representation of DEGs in the form of volcano plot developed in Galaxy software is provided in Figure 2. The hierarchical clustering of DEGs and a heatmap developed in R software is shown in Figure 2. Data analysis and visualization provided in Table 1 and Table 2, Figure 2 are based on comparison analysis between 2mM metformin treatment and control group. Since we found the greatest number of DEGs in response to 2 mM metformin, this dataset was used for further downstream analysis using IPA and g: Profiler software. First, we performed a gene enrichment analysis to identify the overall functions associated with the DEGs. The g: Profiler uses a species-specific genome database, in this case, Gallus gallus, to identify multiple sources of functional evidence, including Gene Ontology (GO) terms, biological processes (BP), regulatory motifs of transcription factors, microRNAs, and protein-protein interaction. The Ensemble gene ids (ENSG) of 365 DEGs were used as input genes in an ordered query (order of decreasing importance based on FDR value) in g: Profiler for statistical enrichment analysis with (g: GOSt). We identified molecular function (MF; Padj 3.400 $\times \;10^{-12}$), biological process (BP; Padj 4.776 $\times \;10^{-6}$), cellular components (CC; Padj 1.452 $\times \;10^{-4}$) and Kyoto Encyclopedia of Genes and Genomes (KEGG) pathway (Padj 1.817 $\times \;10^{-5}$) as the top GO terms based on the expression pattern of dataset molecules (Figure 3). Our analysis using g: GOSt revealed multiple pathways within each GO term; BP, KEEG pathways, MF, and CC are significantly associated with DEGs (Supplementary Table S2).

### 3.3. Identifying the Pathways Affected by Metformin Treatment Using Ingenuity Pathway Analysis (IPA)

The 365 DEGs in COVCAR cells in response to 2mM metformin treatment compared to control group were used for IPA analysis. An analysis setting in IPA for pathway activity of |log2FC| > 1.0 and FDR <0.05 resulted in 203 analysis-ready molecules. The |log2FC| was also used to calculate the directionality (z-scores) of pathways. IPA analysis predicted the activation/inhibition of several pathways. Pathways relevant to the effect of metformin on ovarian cancer cells and those that have biological implications such as AMP-activated protein kinase (AMPK) signaling pathway, epithelial–mesenchymal transition (EMT), angiogenesis, apoptosis, and DNA-damage repair pathways were analyzed using IPA to characterize upstream regulators, causal networks, and canonical pathways.

#### 3.3.1. AMPK Signaling in Ovarian Cancer Cells

**Figure 4:**
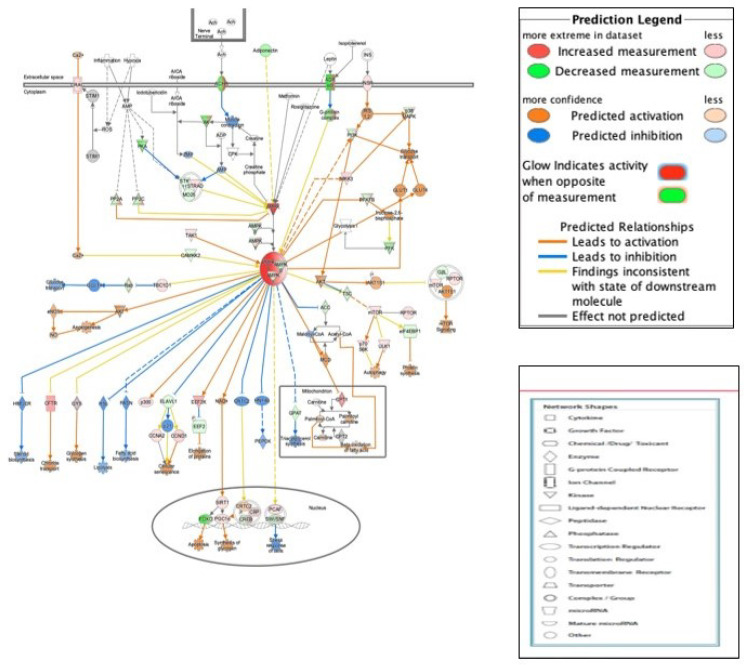
Effect metformin on the AMPK signaling pathway predicted by IPA: the canonical AMPK signaling pathway was overlaid with dataset molecules to predict the downstream effect of AMPK activation. Predicted activation or inhibition of pathways is based on the relationships in the molecular network that represent experimentally observed association between genes and their functions in addition to the literature evidence in the IPA database.

Metformin activates the AMPK pathway to cause phenotypic changes such as reduction in cell proliferation and sphere formation. Hence, in IPA analysis, the canonical AMPK signaling pathway was overlaid with our dataset molecules, and the MAP was turned on to predict the downstream effect of AMPK activation influenced by up and downregulated dataset molecules. The activation of AMPK signaling caused a predicted inhibition of various downstream pathways such as fatty acid biosynthesis, cholesterol biosynthesis, and glucose transport acting through dataset molecules (Figure 4). The activation of AMPK signaling also caused activation of downstream pathways such as apoptosis and elongation of proteins acting through dataset molecules (Figure 4).

#### 3.3.2. Epithelial Mesenchymal Transition

**Figure 5:**
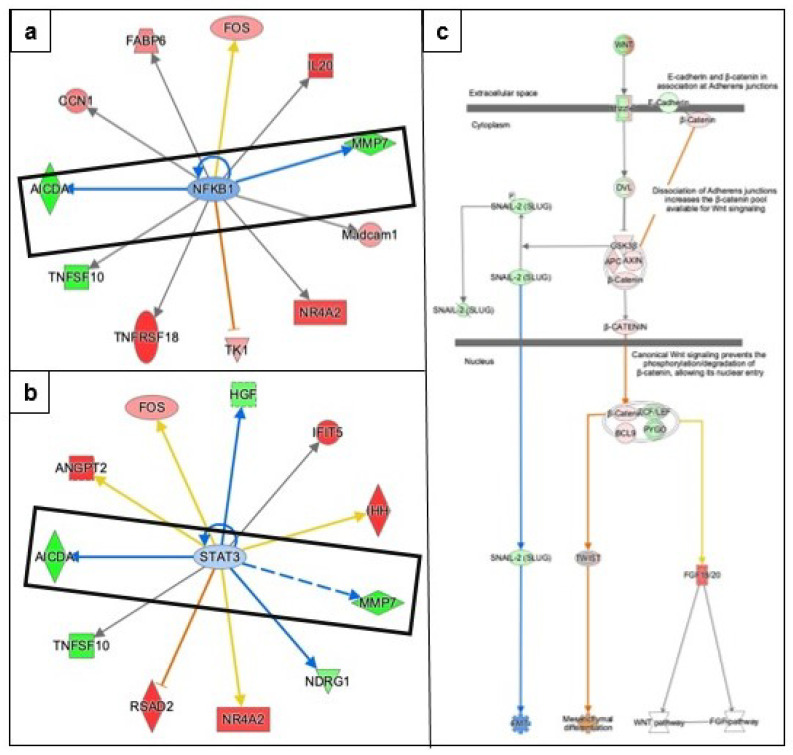
Effect of metformin on epithelial–mesenchymal transition predicted by IPA. Up-stream analysis in IPA predicts the activation/inhibition of various transcription factors. (**a**,**b**) Transcription factors involved in EMT, NFKB, and STAT3 are predicted to be inhibited due to the downregulation of MMP7 and AICDA. (**c**) Canonical WNT signaling pathway involved in EMT was overlaid with dataset molecules that lead to a predicted inhibition of EMT acting downregulation of SNAIl-2 (SLUG). The key for the shape, color, direction, and molecule types in the above pathways is provided in Figure 4.

Epithelial-mesenchymal transition is involved in tumorigenesis and metastasis of ovarian cancer. IPA predicted the inhibition of two transcription regulators: (i) nuclear factor-kappa B (NF-KB) and (ii) signal transducer and activator of transcription 3 (STAT3) involved in EMT (Figure 5) acting through metalloproteinases 7 (MMP7; Log2FC −5.4, FDR 0) and activation-induced cytidine deaminase (AICDA; Log2FC −4.8, FDR 0.0247), the two-top down-regulated genes in response to metformin treatment (Table 1). IPA analysis of canonical Wnt signaling pathway predicted the inhibition of EMT acting through downregulated molecule snail family transcriptional repressor 2 (SNAI1). This pathway also predicted the activation of mesenchymal differentiation affecting through upregulation of beta catenin and downregulation of E-cadherin, acting through transcription regulator twist family BHLH transcription factor 1 (Figure 5). The mechanistic network NFKB is predicted to be inhibited through 59 dataset genes downstream of regulators and 10 transcription regulators and detailed associations between dataset molecules and top transcription regulators are provided in Supplementary Figure S1 and Table S3.

#### 3.3.3. Angiogenesis

**Figure 6:**
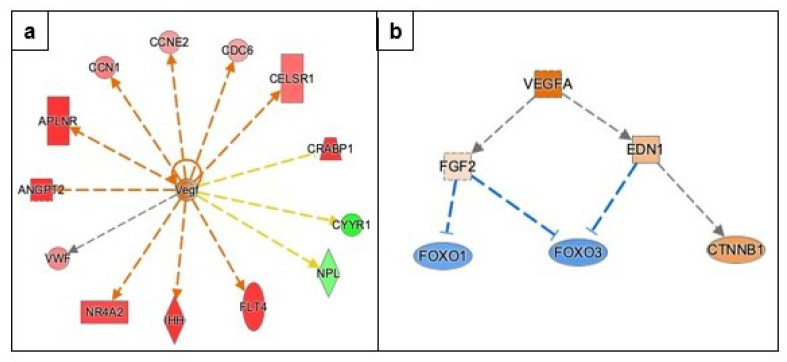
Effect of metformin on angiogenesis as predicted by IPA. (**a**) Transcription factor VEGF involved in angiogenesis is predicted to be activated through the upregulation of molecules in the dataset. (**b**) VEGFA network, the top mechanistic network to be activated acting through *FGF2*, *EDN1*, *FOXO1*, *FOXO3*, and *CTNNB1* regulators. The key for the shape, color, direction and molecule types in the above pathways is provided in Figure 4.

Vascular endothelial growth factors (VEGF) A, B, and C are considered important molecules in tumor angiogenesis. IPA predicted that VEGF, an evolutionarily conserved molecule, is activated with a significant z-score (z-score 2.228, overlap *p*-value 6.45 $\times \;10^{-3}$), and ten of the thirteen genes have a measurement and direction consistent with activation of VEGF (Figure 6 and Supplementary Table S4). The genes activating VEGF are Indian hedgehog (IHH; Log2FC 4.5, FDR 0.0254), angiopoietin 2 (ANGPT2; Log2FC 3.9, FDR 0.0002), Fms related receptor tyrosine kinase 4 (FLT4; Log2FC 2.7, FDR 0.0028). The molecules listed above are also the top upregulated genes in response to metformin treatment (Table 2 and Table S4). The top mechanistic network that is predicted to be activated is the VEGFA mechanistic network (Figure 6). This mechanistic network analysis predicts that VEGFA can indirectly inhibit Forkhead proteins such as FOXO1 and FOXO3 (Figure 6). The genes involved in the VEGFA mechanistic network are provided in Supplementary Table S4.

#### 3.3.4. Regulation of Apoptosis, DNA Damage Repair, and Cell-Cycle Progression

**Figure 7:**
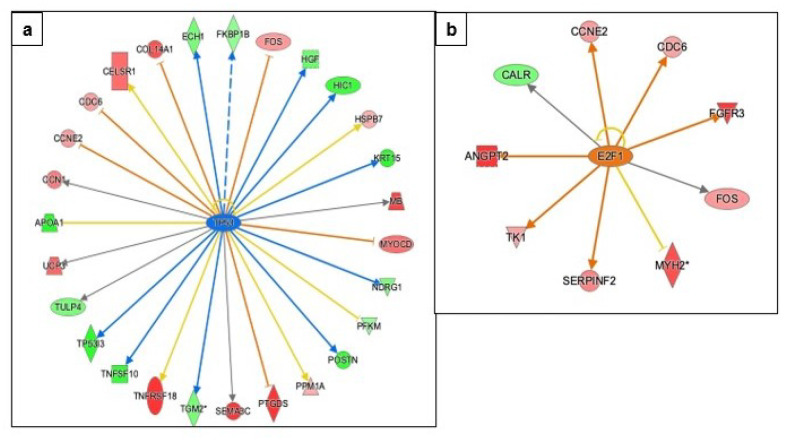
Effect of metformin on apoptosis and cell-cycle regulation as predicted by IPA. (**a**) IPA prediction of upstream regulators revealed that transcription factor TP53 is predicted to be inhibited by the downregulation of dataset molecules. (**b**) Transcription regulator E2F1 is predicted to be activated via upregulated dataset molecules. The key for the shape, color, direction and molecule types in the above pathways is provided in Figure 4.

The TP53 gene is one of the primary molecules involved in apoptosis and DNA-damage-repair pathways in cells. Greater than 50% of all ovarian cancer is associated with mutations in the TP53 gene [25]. IPA predicted the inhibition of TP53 (z-score −2.018, overlap *p* = 1.53 $\times \;10^{-2}$), and 16 of 27 genes have measurement and direction consistent with inhibition of TP53 (Figure 7, Supplementary Table S5). Another transcription factor, E2F transcription factor 1 (E2F1), which plays a role in TP53-mediated apoptosis cell cycle progression, and cell proliferation, is predicted to be activated (z-score 1.720, overlap *p* = 3.92 $\times \;10^{-2}$), and six of the eight genes have measurement and direction consistent with activation of E2F1 (Figure 7). A detailed association between upregulated molecules in the dataset and E2F1 is provided in Supplementary Table S6.

#### 3.3.5. Causal Network Analysis to Identify Master Regulators Associated with Ovarian Cancer

**Figure 8:**
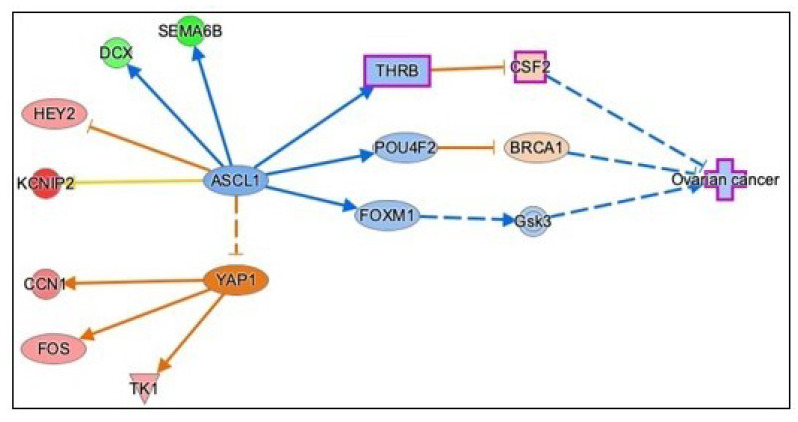
Effect of metformin on ovarian cancer as predicted by causal network analysis in IPA. Causal network analysis connects upstream regulators and dataset molecules in addition to IPA-knowledge-based literature to identify root regulators associated with ovarian cancer. ASCL1 is the master regulator predicted to be inhibited while YAP1 is predicted to be activated, due to downregulation of DCX and SEM6AB and upregulation of CCN1, FOS, and TK1, respectively. The key for the shape, color, direction and molecule types in the above pathways is provided in Figure 4.

We performed causal network analysis in IPA to identify associations among dataset molecules, transcription regulators, and disease processes. Our analysis identified Achaete-Scute family BHLH transcription factor 1 (ASCL1; z-score −2.121, overlap *p* = 6.27 $\times \;10^{-4}$) as the master regulator predicted to be inhibited and thereby causing inhibition of ovarian cancer by six different paths involving transcription regulators such as thyroid hormone receptor beta (THRB), forkhead box protein M1 (FOXM1), POU class 2 homeobox 1 (POUF2), and neuropeptide Y (NYP) in each of the different pathway (Figure 8 and Supplementary Table S7). The down-regulated genes doublecortin (DCX; Log2FC −1.8, FDR 0.0032), and semaphorin 6B (SEMA6B) (Log2FC -3.1, FDR 0.0107), and upregulated gene hairy and enhancer of split (HES) and HES related with YRPW motif protein 2 (HEY2; log2FC 1.4, FDR 0.0489) are responsible for upstream inhibition of ASCL1 and, in turn, ovarian cancer (Figure 8 and Supplementary Table S7). ASCL1 is predicted to inhibit another master regulator, yes-associated protein 1 (YAP1), a transcription regulator known to be involved in malignant ovarian carcinoma. YAP activation is through three upregulated dataset molecules: FOS, thymidine kinase 1 (TK1), and cellular communication network factor 1 (CCN1).

#### 3.3.6. Identification of Gene Networks Associated with Diseases and Cell Function

We identified the top diseases and cell functions activated or inhibited using IPA (Supplementary Table S8). The disease or function involving the greatest number of molecules (124) is ‘tumorigenesis of epithelial neoplasm’ is inhibited (z-score −1.691, *p* = 0.0000797). The disease or function predicted to be activated with the greatest number of molecules (57) is cell movement (z-score 1.994, *p*-value, 0.000025). The top five gene networks based on the expression pattern of DEGs related to cellular and biological processes are provided in Supplementary Table S8.

#### 3.3.7. Identification of Biomarkers Using Bioprofiler in IPA

We performed a Bioprofiler comparison analysis between ovarian cancer signaling pathway and upstream regulators predicted to be activated through dataset molecules, in IPA. We identified ANGPT2 (logFC 3.9, FDR 2.00 $\times \;10^{-4}$), APOA1(logFC −4.4, FDR 0.0067), FOS (logFC 1.4, FDR 0.0059) and VEGFA (z-score 2.228, overlap *p*-value 6.45 $\times \;10^{-3}$) as the molecules of interest that have biomarker applications in various cancers. Both VEGFA and ANGPT2 have been used as biomarkers for measuring the efficacy of various drugs used in cancer treatment. APOA1 mRNA expression has been used as a biomarker for diagnosis and longer survival rates of ovarian cancer [26].

## 4. Discussion

We report here, for the first time, the changes in the transcriptomic profile of primary ovarian cancer cells in response to metformin treatment. COVCAR cells are obtained from the ascites of laying hens that naturally developed epithelial ovarian adenocarcinoma. We found that metformin treatment of COVCAR cells induced a significant up-regulation of genes involved in apoptosis, such as tumor necrosis factor receptor super-family member 18 (TNFRSF18), ubiquitin associated and SH3 domain containing A (UBASH3A) and dexamethasone-induced Ras-related protein 1 (RASD1) acting through (ERK) gene network.

Our analysis shows that at 0.5 mM and 1mM metformin treatment shows a significant biological effect that is not reflected at the genome level. Whereas metformin treatment at 2mM shows a significant effect at both biological and genome level. This discrepancy can be due the non-genomic effects of metformin. The difference between phenotypic effect and gene expression can also be explained by data analysis used in transcriptome studies. RNASeq data analysis using DESeq2 considers false discovery rate (FDR) for significance in order to remove false positive genes and as a result some of the significant genes at *p* < 0.05 might be eliminated when FDR is set at <0.05.

Our pathway analysis using IPA revealed that metformin treatment affects both AMPK-dependent and AMPK-independent pathways. Our findings are consistent with a previous report that metformin treatment of several ovarian cancer cell lines activates both AMPK-dependent and AMPK-independent pathways [14]. IPA prediction analysis showed several AMPK-dependent inhibitions of pathways that are involved in lipid homeostasis. Some of them include downregulation of acetyl-CoA carboxylase (ACC) and glycerol 3-phosphate acyltransferase (GPAT) that could potentially lead to a decrease in fatty acid and triglyceride synthesis. Our IPA prediction analysis showed inhibition of lipolysis via hormone-sensitive lipase (HSL), steroid biosynthesis via 3-hydroxy 3-methylglutaryl-CoA reductase (HMGCR), and gluconeogenesis via phosphoenolpyruvate carboxykinase (PECK). We found that metformin treatment is likely to affect glucose transport via the AMPK-dependent pathway in COVCAR cells by inhibiting glucose transporter type 4 (GLUT4). In contrast, GLUT1 and GLUT4 are predicted to be upregulated as a compensatory mechanism through activation of the insulin receptor signaling pathway.

We found another AMPK-dependent pathway-related activation of apoptosis via downregulation of FOXO protein. This activation is likely due to the upregulation of the Sirtuin family of NAD+-dependent deacetylases (SIRT1) as found in our analysis. SIRT1 has been found to rescue cells from oxidative-stress-induced apoptosis by promoting ubiquitination and degradation of FOXO [27,28] but in cancer cells these pathways could be dysregulated [29], resulting in activated apoptosis. Although our analysis did not predict activation of a pathway between SIRT1 and FOXO, both SIRT1 and SIRTI2 deacetylates FOXO and regulates cell processes involved in cell proliferation, oxidative stress, and apoptosis [27,30].

Our pathway analysis reveals multiple AMPK-independent pathways via activation or inhibition of upstream regulators and gene networks in response to metformin treatment in COVCAR cells. One such AMPK-independent pathway is the down-regulation of extracellular-signal-regulated kinase (ERK) (Supplementary Figure S1) signaling pathway, an observation consistent with previous report that suggests metformin treatment of HO-8910 human ovarian cancer cell lines inactivate ERK1/2 and thereby reduce cell viability and induce apoptosis in a concentration-dependent manner [31].

We found that metformin treatment of COVCAR cells induced a significant up-regulation of molecules such as tumor necrosis factor receptor super-family member 18 (TNFRSF18), ubiquitin associated and SH3 domain containing A (UBASH3A) and dexamethasone-induced Ras-related protein 1 (RASD1) involved in the (ERK) gene network [Supplemental Figure S2]. RASD1 is involved in the apoptosis of prostate cancer cells treated with an anti-cancer drug, formononetin [32] and in breast cancer cells treated with calycosin [33]. Another upregulated molecule in our dataset TNFRSF18 is activated upon T cell activation and induces apoptosis by binding to the pro-apoptotic molecule CD27BP55 [34]. An upregulated gene transcript in the ERK pathway, UBASH3A is a negative regulator of T-cell activation and function [35] and can induce caspase-dependent apoptosis [36]. This is the first report to document significant upregulation of critical apoptosis-inducing molecules such as RASD1, TNFRSF18, and USBASHA in response to metformin treatment. Based on the foregoing, we hypothesize that metformin is likely to induce apoptosis in COVCAR cells leading to decreased sphere formation. Such molecules could, therefore, be targeted for inducing apoptosis in ovarian cancer cells.

We found that metformin treatment of COVCAR cells resulted in a predicted inhibition of epithelial to mesenchymal transformation (EMT). EMT is a tightly regulated process in which epithelial cells lose their normal interaction with the basement membrane and undergo multiple cellular changes to convert into mesenchymal phenotype [37,38]. Our pathway analysis revealed predicted inhibition of NFKB and STAT3, the two transcription factors involved in EMT via downregulation of molecules such as MMP7 and AICDA. Single-cell EMT-related transcriptomic analysis of cells derived from ascites of women with epithelial ovarian cancer revealed a heterogeneous cluster of genes involved in promoting EMT [39] indicating that inhibition of EMT is a essential step in reducing tumorogenesis.

Metformin treatment of SKOV3 and primary breast cancer cells has been shown to inhibit EMT. In addition, metformin treatment of SKOV3 cells significantly reduced interleukin 6 (IL-6), an EMT-inducing factor, by affecting NFKB and STAT3 signaling pathway [40]. Furthermore, metformin treatment of two primary breast cancer cells MBCDF and MBCD17 downregulated mesenchymal signature genes vimentin and SNAIL and inactivated NFKB and STAT3 signaling pathways in response to IL-6 [41]. In the same study, NFKB inactivation was independent of AMPK signaling, whereas the inactivation of STAT3 depended on the activation of the AMPK signaling pathway. Based on the foregoing, metformin treatment of COVCAR cells is likely to inhibit EMT through both AMPK-dependent and AMPK-independent pathways.

Our IPA data analysis indicates that metformin treatment of COVCAR cells could potentially lead to upregulation of transcriptomic pathways involved in angiogenesis likely due to the activation of AMPK. The predicted activation of VEGFA is probably the result of significant up-regulation of genes involved in angiogenesis such as ANGPT2, FLT4, apelin receptor (APLNR), nuclear receptor subfamily 4 (NR4A2). The activated angiogenesis pathway in our analysis using IPA is consistent with previous studies using breast cancer cell lines and melanoma cells treated with metformin. A greater VEGF expression at mRNA and protein levels was observed in the estrogen receptor alpha -negative MDA-MB-435 breast cancer cell line treated with metformin in an AMPK-dependent manner [42]. Metformin treatment of BRAF-mutant melanoma cells was found to upregulate VEGF expression in an AMPK- dependent manner [43]. On the contrary, metformin treatment of human ovarian cancer cell line HO-8910 significantly decreased VEGF and VEGFR2 at mRNA and protein levels in a concentration-dependent manner [31].

While VEGF and VEGFR play an essential role in ovarian cancer progression, recent studies have shown that promoters of angiogenesis such as angiopoietins, apelin receptors, and its ligand apelin, NR4A2, also play an equally important role. The upregulation of ANGPT found in our RNASeq analysis is consistent with studies using mouse models wherein an increased expression of angiopoietins (ANGPT1, ANGPT2, and ANGPT4 and TIE2 (angiopoietin receptor) have been shown to promote ovarian cancer progression [44]. ANGPT2, secreted by activated endothelial cells, facilitates VEGF-dependent angiogenesis by inducing vascular destabilization leading to leaky vasculature. The molecular mechanism involving VEGF-ANG- TIE2 pathway is an essential regulator of tumor angiogenesis [45].

Our RNASeq data analysis revealed a significant upregulation of APLNR (log FC 3.4, FDR 0.0018). APLNR and its ligand apelin are involved in promoting tumor angiogenesis via multiple autocrine and paracrine mechanisms [45,46,47]. In support of our findings, several ovarian cancer cell lines such as OVCAR-3, -4, -5 and SKOV3 and high grade serous ovarian carcinoma (HGSOC) tissue were found to have greater expression of mRNA and protein of APLNR and its ligand apelin as compared to normal ovarian surface epithelium [48]. In addition, a meta-analysis of 16 ovarian cancer cell lines and ovarian tumors suggested a significantly elevated APLNR expression that is correlated with poor prognosis in patients with serous ovarian cancer [48].

Our IPA data analysis revealed a mechanistic network that links VEGF activation to inhibition of forkhead transcription factors (FOXO). Two of the FOXO family members are involved in angiogenesis and neovascularization via activation of angiopoietins and FLT4 [49]. FOXO3 is also known to repress VEGF signaling via miRNAs [50]. Overall, pathways involved in angiogenesis were found to be activated in response to 2 mM metformin treatment.

Our analysis indicated that metformin treatment of COVCAR cells resulted in IPA-predicted inhibition of TP53 and activation of E2F1, which are involved in cell-cycle progression, DNA-damage repair, and apoptosis. Our analysis of IPA-predicted inhibition of TP53 is consistent with the studies on hepatocellular carcinoma cells (HCC) that showed metformin downregulated TP53 via through AMPK-SIRT1 pathway in the presence of high glucose [50]. On the contrary, metformin can activate TP53 by phosphorylating AMPK, and in turn cause cell cycle arrest and apoptosis in cancer cells [51,52]. Although available evidence points to the activation of TP53 via AMPK in cancer cells, it should be noted that most cancers have lost or mutated TP53 [53] which can lead to dysregulation in the TP53 signaling pathway.

Our pathway analysis suggests that the predicted activation of E2F1 is through upregulation of ANGPT2, cell cycle division 6 (CDC6), and fibroblast growth factor receptor 2 (FGFR2) that are involved in cell proliferation [54], cell cycle division and DNA replication [55]. E2F1 was found to induce pro-proliferation genes or pro-apoptosis genes in response to high and low levels, respectively, thereby either stimulating cancer growth or improving the overall prognosis of ovarian cancer [56]. The apoptosis-inducing effect of E2F1 could be mediated via TP53-dependent manner [57] or TP53-independent manner [55], by activating pro-apoptotic genes [58].

The IPA software was used to perform causal network analysis (CNA) for identifying novel master-regulators by creating pathways of literature-based relationships. We performed CNA using our RNASeq dataset to identify master regulators that are associated with ovarian cancer. Our analysis suggests/predicts that metformin treatment is associated with ‘inhibition of ovarian cancer’ through the master regulator, ASCL1. This master regulator is predicted to inhibit ovarian cancer by acting through 6 pathways. One of the pathways involved is through inhibition of FOXM1 and GSK3. Integrated genomics analysis indicates that FOXM1 transcription factor network is significantly altered in 87% of cases of ovarian cancer [25]. Interestingly, TP53 represses FOXM1 following DNA damage [58] suggesting that the high rate of TP53 mutation in high-grade serous ovarian cancer contributes to FOXM1 over-expression. The master regulator, ASCL1, inhibits the activation of YAP1, a transcription factor, that is over-expressed at both mRNA and protein levels in ovarian cancer. YAP1 promotes EMT, cancer growth, and tumorigenesis [59]. YAP expression levels are positively correlated with TEAD4 (the c-terminal region of transcriptional enhancer factor TEF-1) levels [60] and their co-expression is a prognostic marker for poor ovarian cancer survival [59]. Overall, the master regulator, ASCL1, is involved in various pathways that can lead to the predicted inhibition of ovarian cancer.

## 5. Conclusions

In summary, we provide novel evidence that metformin treatment leads to significant changes in the phenotype and the transcriptome of ovarian cancer cells obtained from the leghorn chicken model of ovarian cancer. We report for the first time the use of spheres from COVCAR cells for transcriptomic analysis. The advanced pathway analysis performed using IPA revealed several novel target genes in addition to predicting gene network pathways and transcription regulators that are likely to be influenced by the metformin treatment of ovarian cancer cells. The gene signatures obtained from the RNA-seq analysis of COVCAR cells are consistent with an integrated genomic analysis of human ovarian cancer. The leghorn chicken model of ovarian cancer, therefore, is an appropriate animal model both at the organismal and genomic level to study ovarian cancer. Future investigations using our transcriptomic data would lead to understanding molecular mechanisms of metformin action, to identify biomarkers and drug targets for ovarian cancer therapy or prevention.

## Figures and Tables

**Table 1:** Top 10 mRNAs that are downregulated in COVCAR cells in response to 2 mM metformin treatment compared to control as detected by RNA-seq analysis. Functions listed below are that of the protein encoded by the respective gene. FDR-False discovery Rate.

Gene Name	Log2FC	*p*-Value	FDR	Function
ABHD8	−6.5	6.12 $\times \;10^{-5}$	7.60 $\times \;10^{-3}$	Influences methylation of 12CpG and increased EOC risk
MMP7	−5.4	6.07 $\times \;10^{-8}$	0.00	An enzyme involved in extracellular matrix lysis
APOC3	−5.3	1.55 $\times \;10^{-6}$	6.00 $\times \;10^{-4}$	Involved in the metabolism of triglyceride (TG)-rich lipoproteins (TRLs)
AICDA	−4.8	3.93 $\times \;10^{-4}$	2.47 $\times \;10^{-2}$	Involved in somatic hypermutation, gene conversion, and class-switch recombination of immunoglobulin genes in B-lymphocytes
GDPD2	−4.5	2.28 $\times \;10^{-5}$	3.80 $\times \;10^{-3}$	Plays a role in the remodeling of the actin cytoskeleton
APOA1	−4.4	5.28 $\times \;10^{-5}$	6.70 $\times \;10^{-3}$	Attaches to cell membranes and promotes the efflux of cholesterol and phospholipids from the cytoplasm
CYYR1	−4.2	8.24 $\times \;10^{-4}$	4.01 $\times \;10^{-2}$	Not annotated in the Gallus gallus genome
GSTA4	−3.9	1.30 $\times \;10^{-4}$	1.20 $\times \;10^{-2}$	Associated with hepatocellular carcinoma
S1PR3	−3.7	1.96 $\times \;10^{-5}$	3.50 $\times \;10^{-3}$	A G-protein-coupled receptor that contributes to the regulation of angiogenesis and vascular endothelial cell function

**Table 2:** Top 10 mRNAs that are upregulated in COVCAR cells in response to 2 mM metformin treatment compared to control as detected by RNA-seq analysis. Functions listed below are that of the protein encoded by the respective gene. FDR—false discovery rate.

Gene Name	Log2FC	*p*-Value	FDR	Function
MNR2	5.5	1.85 $\times \;10^{-5}$	3.40 $\times \;10^{-3}$	Regulation of transcription, sequence specific-DNA binding
SLC5A5	5.5	2.47 $\times \;10^{-4}$	1.85 $\times \;10^{-2}$	A member of the sodium-glucose co-transporter family. Responsible for the uptake of iodine in thyroid and lactating breast tissue
TMEM82	4.7	2.37 $\times \;10^{-6}$	8.00 $\times \;10^{-4}$	Transmembrane protein. Prognostic marker in Renal cancer (favorable)
IHH	4.6	4.15 $\times \;10^{-4}$	2.54 $\times \;10^{-2}$	Activation of the hedgehog pathway, implicated in the development of various cancers, prostate cancer, pancreatic cancer, gastrointestinal malignancies, and ovarian cancers
IL19	4.5	3.32 $\times \;10^{-4}$	2.19 $\times \;10^{-2}$	Mitogenic and chemotactic for endothelial cells and can induce their angiogenic potential
UNC45B	4.2	5.45 $\times \;10^{-4}$	3.04 $\times \;10^{-2}$	Encodes a myosin-specific chaperone that, together with the general heat-shock protein HSP90, is involved in myosin assembly
CHRNB3	4.2	1.01 $\times \;10^{-3}$	4.61 $\times \;10^{-2}$	Not annotated in the Gallus gallus genome
CRABP-I	4.1	6.50 $\times \;10^{-4}$	3.39 $\times \;10^{-2}$	CRABP1 is assumed to play an important role in retinoic acid-mediated differentiation and proliferation processes
RSAD2	4	3.29 $\times \;10^{-6}$	1.10 $\times \;10^{-3}$	Induced by interferon, known to abolish amino acid and mitochondrial metabolism
SLC6A17	4	3.80 $\times \;10^{-16}$	0.00	A member of the SLC6 family of transporters, responsible for the presynaptic uptake of most neurotransmitters

## Data Availability

RNASeq data available through, GEO Accession: GSE182084. https://www.ncbi.nlm.nih.gov/geo/query/acc.cgi?acc=GSE182084, accessed on 1 October 2021.

## Supplementary Materials

The following are available online at https://www.mdpi.com/article/10.3390/genes13010030/s1, Table S1: List of 365 DEGs in 2 mM metformin-treated group as compared to the control group, FDR < 0.05, analyzed using DESeq2, R V.4.1.0.; Table S2: Identifying gene ontology terms (GO) in g: Profiler. Statistical enchrichment analysis usign g:GOst of 365 significant DEGs in the form of ordered query, padj < 0.05; Table S3: NFKB mechanistic network of association between upstream regualators and dataset genes, created in IPA; Table S4: VEGF mechanistic network of association between upstream regualators and dataset genes, created in IPA; Table S5: All genes associated with the predicted inhibition of TP53; Table S6: All genes associated with the predicted activation of E2F1; Table S7: Paths through which master regulator ASCl1 can affect ovarian cancer; Table S8: Top 5 diseases and function predicted to be activated and inhibited; Figure S1: Effect metformin on the NFKB1 mechanistic network as predicted by IPA; Figure S2: Effect metformin on the ERK signaling pathway predicted by IPA.
