# Supplementary figures and images for: Metformin Affects the Transcriptomic Profile of Chicken Ovarian Cancer Cells
Source: Genes (Basel). 2021 Dec 23;13(1):30. doi: 10.3390/genes13010030 (PMC8774788; doi:10.3390/genes13010030)

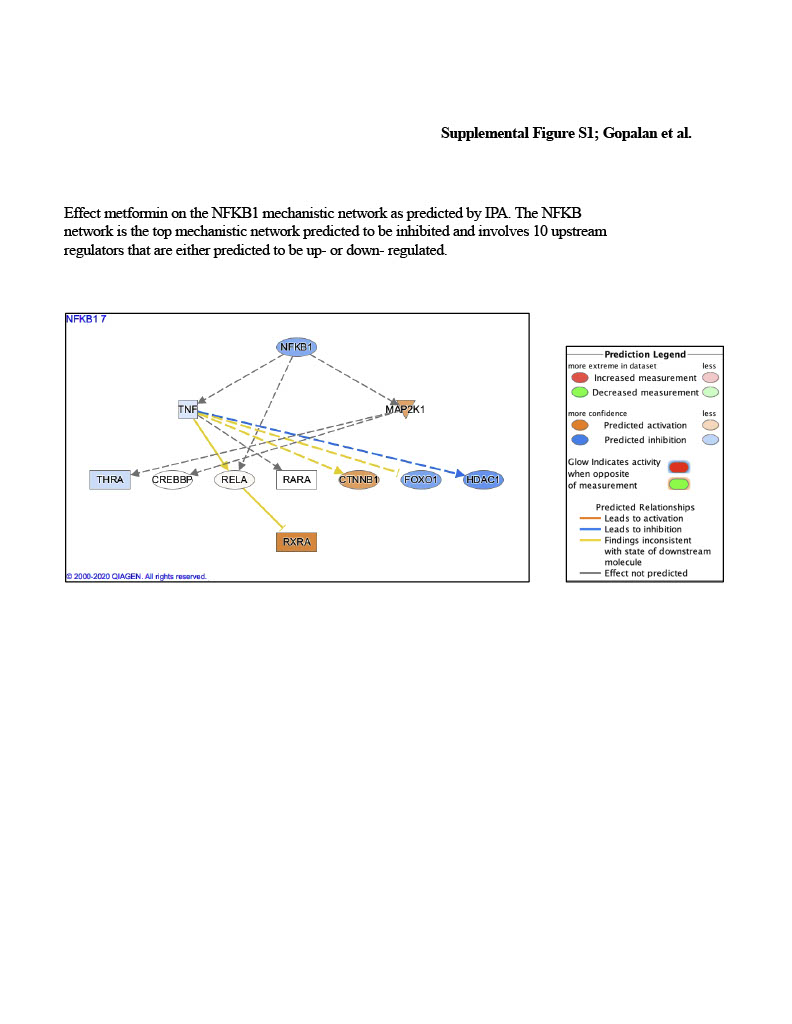

Supplement: Supplementary file 1 [file genes-13-00030-s001.zip › Supplementary Figure S1.jpg]

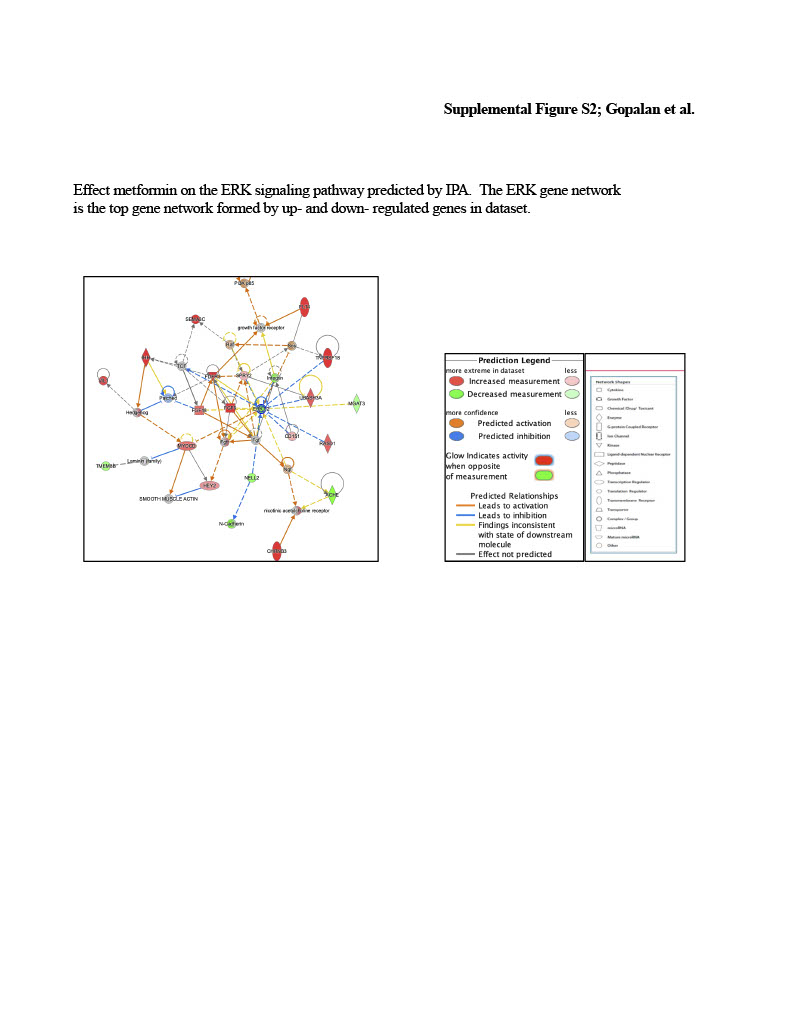

Supplement: Supplementary file 1 [file genes-13-00030-s001.zip › Supplementary Figure S2.jpg]
